# It’s better to run towards the fire: The experience of reserve duty for reservists with PTSD prior to re-enlistment

**DOI:** 10.1016/j.ijchp.2025.100621

**Published:** 2025-09-20

**Authors:** Anna Harwood-Gross, Shir Elias, Dalia Amit Zivan, Rona Davis

**Affiliations:** a METIV Israel Psychotrauma Center, Herzog Medical Center, Israel; b Department of Psychiatry, Leiden University Medical Center, the Netherlands

**Keywords:** PTSD, Military, Veterans, Occupational functioning, Ongoing trauma, Moral injury

## Abstract

**Background:**

Little research exists delineating the experience of serving in the military with PTSD despite longitudinal studies indicating that a small percentage of deployed combat soldiers have PTSD prior to deployment. Following a mass re-enlistment of reserves, during the Iron Swords war, the current qualitative study aimed to describe the experience of fifteen reservists with pre-existing PTSD and explore the clinical difficulty or utility of their service.

**Methods:**

Reservists were interviewed by trained clinicians and interviews were transcribed and analyzed using a grounded descriptive phenomenological approach.

**Results:**

Findings demonstrated a key theme of a reparative experience in addition to the differentiation between functioning on the domestic and military front and the differentiation between the fantasy of success versus the reality of re-enlistment with PTSD. The experience of re-enlistment as a reservist compared to the original PTSD-inducing service was described in terms of enhanced capability, a greater awareness of emotional needs by seniors and the establishment in general, enhanced choice (including that to re-enlist) and the utility of PTSD symptoms such as hyperawareness, on the battlefield compared to the futility at home.

**Conclusions:**

The study highlighted the unknown nature of repeated duty and the potential for increased difficulty of transition between military and domestic spheres while acknowledging the potential for a positive experience of re-enlistment with PTSD. The findings reinforced the need for clear clinical guidelines within the military and importance of monitoring for risk of deterioration of symptoms both on the battlefield and following discharge when enlisting those with PTSD.

## Introduction

The prevalence of PTSD amongst veteran soldiers, both combatant and non-combatant, is well documented (Hines et al., 2014) but there are indications anecdotally (Levinson, 2025) and embedded in longitudinal research samples, that soldiers with clinically diagnosed PTSD are sent on active deployment. PTSD rates amongst soldiers in active deployment have been cited as low as 0.6 % (Garg & Mishra, 2024) though this figure does not align with longitudinal studies. A longitudinal study on British combatants in the 2003 Iraq war reported that 2–4 % of those currently in service displayed a tentative PTSD diagnosis at time of testing even at baseline. It is unclear how many were in active deployment or combat at the various time points though personal correspondence indicated a mean rate of 2 % of active servicemen (Hotopf et al., 2006). In cohort studies of combatants who were subsequently dispatched to and returned from Iraq, 16 % of participants at pre-deployment had suspected PTSD (self-report) (Wright et al., 2012) and of the 12 % of Dutch infantry troops returning from Iraq with clinically diagnosed PTSD, around half of them reported that PTSD symptoms were from previous life events or deployments (Engelhard et al., 2007). In a limited population study, reservists who had recovered from PTSD were re-enlisted and demonstrated to have increased PTSD vulnerability following re-enlistment (Solomon et al., 1990).

On October 7, 2023, Israel was attacked when thousands of Hamas terrorists entered from its Gaza border, while rockets were fired from the northern and the southern borders. During this unprecedented event, hundreds of thousands of reservists were summoned to reserve duty and cars full of returning soldiers who had not yet been drafted were reported to be lined up in kilometers-long lines outside army bases (Hendrix et al., 2023; Surkes, 2024). Among the population of reserve soldiers, as an outcome of Israel’s defense history in recent decades, many of the draftees had previously served in major operations.

Medically and psychologically, deployment with PTSD is not promoted. Antecedent PTSD symptoms have frequently been associated with greater levels post deployment PTSD symptoms (Wald et al., 2013) and many biological predictors of PTSD development following trauma are those also associated with previous trauma exposure, such as heightened arousal and reduced regulation (Zoladz & Diamond, 2013). Indeed, during the write up of the current study, the news of recruitment of soldiers with PTSD was revealed in the national press and criticized by psychiatry and suicide experts (Levinson, 2025). While active duty with PTSD symptoms may be a risk factor for increased symptoms following deployment, there is clinical reasoning to believe that the outcome may be more complex.

Evidence based treatment of PTSD predominantly centers around confronting avoidance of the trauma reminders to varying degrees of intensity. For example, Prolonged Exposure (PE) one of the leading treatments for PTSD, is based on graded exposure to reduce avoidance and hyperarousal in response to trauma reminders (Foa et al., 2007). The act of returning to war with PTSD, if successful, may serve a therapeutic purpose of confronting avoidance symptoms and regaining self-efficacy, though this would be dependent on a restorative experience. PE, for example, requires exposure in a controlled environment, primarily to reduce the risk of re-traumatization and further dysregulation (Foa et al., 2007).

On the other hand, exposure to new traumatic events, highly likely in repeated warfare, may indeed challenge an already vulnerable and activated or sensitive stress system and reduce the likelihood of successfully regulating following exposure to traumatic stress (Lanius et al., 2010; Nanney et al., 2018). This poses a difficulty on the warfront as a restorative stress system is required to recover from acute combat stress reactions which are common in intense combat situations.

The current study was designed based on clinical findings from a PTSD clinic in Israel. When the Iron Swords war, broke out on October the 7th 2023 it became evident that in light of the new reality, patients being treated in the PTSD clinic were turning to their therapists for a new kind of guidance; whether to enlist, how to maintain their composure on the battlefield and how to utilize their therapy to confront the war on all fronts. Many patients informed therapists that they had been conscripted, many of them to the same units and the same combat zones in which they had sustained their original mental injuries. Some notified therapists of their decisions to conscript despite having retired with mental health injuries, some shared their decision to not comply with the draft and many expressed a difficulty grappling with the dilemma of how to protect their and their families’ psychological wellbeing whilst facing re-enlistment. Therapists anecdotally reported discussions of loyalty to military units, a deep desire to share the burden, a desire for healing alongside fears of further suffering, fear of disintegration and doubts in their ability to endure combat. This clash between inner psychological wounds with a demanding reality, calling for action, hinting at a possibility for repair, prompted the current study.

The study was designed to explore the experience of soldiers who were drafted for reserve duty despite being diagnosed with from PTSD from previous combat. The study was conducted in a unique zeitgeist and reflected a national motivation to speedily generate research projects to understand the unique phenomenon witnessed during a period of unprecedented warfare. The study aimed to understand whether soldiers’ experiences reflected the clinical and scientific indications that returning to the battlefield with PTSD may be both harmful and helpful and to delineate the experience from the soldiers’ perspective.

## Method

Fifteen participants were recruited to the study and interviewed though only twelve participants had their interviews transcribed due to a recording failure in the interview room. The participants were recruited through METIV Israel Psychotrauma Center’s veteran clinic and through flyers that were sent to mental health officers and therapists and were advertised on social media. Two participants were referred to the study by the clinic. According to the grounded theory approach of qualitative analysis (Glaser & Strauss, 1967), interviews were analyzed before the total sample was recruited and recruitment continued until data saturation as outlined in the data analysis section. In total, the transcripts of 12 combat soldiers diagnosed formally or informally with PTSD from a previous combat who served in the “Iron Sword” war, were analyzed (demographics outlined in Table 1). The PTSD inclusion criterion which was preregistered in the study design included: referral from a therapist or mental health officer who evaluated PTSD prior to the reserve duty; treatment for PTSD prior to reserve duty; PTSD diagnosis from a psychiatrist prior to reserve duty; PTSD diagnosis from the Ministry of Defense, or the National Insurance assessment services.

**Table 1:** Demographic characteristics of participants with transcribed interviews (*N* = 12).

Demographic characteristic		Mean (SD) or total
Age		(7.6)38.4
Marital status	Married	6
	Single with a partner	3
	Single	1
	Divorced	2
Number of children	Mean (standard deviation)	(1.8)2.2
Education	High school	2
Academic (BA/BSc)		9
	Academic (MA/PhD)	1
Employment	Employed	10
	Unemployed	2
Diagnosed with PTSD	Recognized with PTSD by MOD	8
	Diagnosed by non-MOD professional	4
Number of years since initial traumatic event		(6.2)17.9
Age at the time initial traumatic event		(1.8)19.5
Drafted into reserves	On 7.10.23	6
	After 7.10.23	6

Interviews occurred on zoom or in person at the outpatient clinic in METIV– the Israel Center for Psychotrauma. The interviews were conducted by certified clinical therapists and recorded and transcribed on approved software. They followed a semi-structured interview schedule and interviewers were free to adjust the interview as needed. Fourteen interviews were in Hebrew and one in English (Michael, pseudonym). Quotes in Hebrew were translated by a professional translator and edited by the bilingual members of the research team. The interview questions are available (appendix A).

The present study was approved by the Institutional Review Board of Bar-Ilan University. All participants signed an informed consent and were free to end the interview at their will. If needed, the participants received information regarding treatment options at the research site or were helped by their interviewer to find appropriate therapy. All names in the following analyses are pseudonyms.

### Data analysis

Data was analyzed using a descriptive phenomenological approach in order to focus the analysis on the essential essence of experience (Todres, 2005). Data analysis was conducted in stages; Upon each individual reading of the text researchers attempted to immerse themselves in the whole experience of the text. Rather than initially coding and developing themes, the primary goal of the first reading was to assess the overall experience of the process of deciding to enlist, enlistment, service and repatriation with PTSD. These general themes were discussed in collaborative meetings and distinct units of meaning or codes (as described in thematic phenomenological analysis) (Braun & Clarke, 2021a, 2021b)were generated. Secondly, each text was then analyzed according to these discrete units of meaning. The units of meanings included longer undefined significant excerpts but also specific codes. These units of meaning across interviews were then discussed via online team meetings and collaborative documentation in order to develop themes or structures which contribute to the understanding of the general phenomenon. Collaborative documentation included describing themes and sub-themes in greater detail and continuously returning to interviews to identify relevant quotes which could be assigned to said themes. In these discussions, the three interviews which were not transcribed were also included in the process. The emerging formulation of the general phenomenon was then assessed and reassessed as a whole and as a sum of the individual parts identified.

### Participants

The 12 participants whose interviews were analyzed were men who identified as Jewish (this reflects the demographics of the Israeli military’s combat units though exact figures cannot be ascertained due to lack of publicly available data). All the participants reported that their primary traumatic event occurred when they were between 19–22 years of age, during their regular military service, some reported additional events when they were on reserve duty. The participants reported variations in the intensities of their post-traumatic symptoms, the frequencies of their occurrence and in the levels of severity of impact on their functionality. They all reported active combat service.

## Results

The experience of reservists with PTSD following the October 7th invasion into Israel and the subsequent mass draft and multiple-front war was experienced as a multi-faceted experience. Three prominent themes were identified as central to the reservists’ experiences with themes reflected upon in both positive and negative frameworks.

The three themes identified were:

1. “Between Spaces” – describing the difference between being a civilian and a soldier, in the domestic and military space.
2. “Then and now” – describing a remedial experience in comparison to their original, PTSD inducing, military experience.
3. “The intersection of fantasy with reality” – describing the gulf between the fantasy of repair versus the complexity of reality

### Between spaces: being a civilian and a soldier, in the domestic and military space

The moment war broke out, participants described a dilemma between the experience of passivity at home compared to a return to the combat zone where they could act and thus experience a sense of purpose or control.

Colin, a 38 year old combat medic explained, “*I found it very difficult to cope with the situation […] when I knew that there is a war and there is nothing for me to do other than sit and watch the news*”. The distinction between the chaos and uncertainty on the home front was compared with the sense of control and order experienced with re-enlistment. Peter, a 46 year old evacuation room commander described a feeling of relief, how only when in the throws of tangible war, could he stop running and actually breathe. This feeling was accompanied by a sense of purpose which he described as being ‘goal oriented’:

*I was awfully, awfully, awfully goal oriented. Actually the first time I felt as though I could breathe was when I arrived at Nahal Oz [a border community which was under attack] to be with the battalion under fire. That means it was the first time I said: OK, that’s it, I can stop running, I can calm down. And now I am on the schedule. I am part of the schedule. It’s really at that level […] you want to be with your people […] I know all the commanding officers, I know most of the soldiers. I was a popular figure in the battalion. I mean I was there for 20 some years after all. So yes, it was a relief when I got there – a big relief.*

This movement between order and disorder was evident for many participants. The home-front was described in terms of difficulty, confusion and consisting of a lack of clear direction. Opposing this experience was the order and clear roles designated within the military sphere. The ability to regain a sense of order through rejoining their units, was perceived as important. Andy, a 49 year old reservist in charge of evacuation of bodies, described this dichotomy:

*I felt as though we are stepping into the unknown. That there is a lack of clarity. And it is frightening. […] I feel, enormous anguish, physically in my body. I have a gut feeling that this is real […] to a certain extent I was trembling, but the big comfort was meeting the people who give you that feeling of confidence under stress when we were actually all helpless, but on the other hand there is this war, and when I get there, our organic team where we are very strongly bonded together, is very comforting, because you return to part of your family, you return to the safe place. Everyone is in a safe place.*

This order, was regained both through a return to familiarity, to a unit comprised of former colleagues, and also to a familiar role that they had never stopped fulfilling. “*I arrived immediately (*on the day war broke out) *[…] and later someone asked me something like: what’s it like to return to being a soldier from being a citizen? I answered very, very simply: I never stopped being a soldier”.* (Andy)

The return to battle for some of these reservists with PTSD, was reflected upon as a return to a reality which had not fully been left behind. Despite acknowledging the absurdity, “the f**kedness of this is clear to me”, Peter explained, “*You see everyone, the visuals are very familiar, the body remembers, I mean the visual of seeing a trail of convoys leaving, people running, you say: OK, “we’re home.”*

For Andy, this return to reserve duty with PTSD, however, was experienced as a double edged sword, as it led him to feel both stress and security simultaneously:

*It (reserve duty) was both my anchor and my hanging rope […]. Look, we always had stressors, there was always […] a difficulty, but at the same time, the only place where I was, free and happy and calm, was in the reserves. No other place, nothing else, gave me the feeling of security, and the feeling of belonging.*

A similar feeling of opposites, that were not easily united, was described by James, a 42 year old missing persons’ officer, who felt a sense of both belonging and of not belonging to civil life and to the battlefield. “*I get home, and I want to be in Gaza, but in Gaza I want to be home. You’re neither here nor there”.*

Tensions, however, were also experienced between mundane and heroic tasks. Gary a 39 year old combat medic, described his inability to settle between his familial fatherly roles and his combat roles. His difficulty in finding meaning in civilian life, had a direct effect on his and his family’s emotional well being:

*And I felt a kind of meaninglessness, that if before this, the health of some 500 soldiers was dependent on my functioning – I suddenly find myself in the living room chasing after a two year-old girl [daughter] […] and this gap between doing things that are crucial and significant for the whole world, and dealing with small daily bothersome human issues, […] because you have no patience – created an enormous gap, a great deal of tension with the wife, and we are just beginning couples therapy, and not in the best state.*

This difficulty was most prominent in participants’ encounter with their children and reflected an overarching theme seen in reservists with and without PTSD. Michael, a 32 year old combat soldier, encapsulated this difficulty, particularly reflecting on the tension between the needs of a civilian father versus the need to process extreme military experiences, *“I'm reconciling things, I'm aware that. I am on the edge as well. I just came from getting shot at and shooting people to managing a baby and two kids, and I sometimes have an outburst at my kids or out my wife”.*

Different to the experiences of reservists without PTSD, the movement between military and civilian realms also offered an added tension, the return to daily reminders of disability and a PTSD diagnosis. Samuel, a 30 year old combat solder reflected on his ability on the battlefield compared to the markers of disability in the civilian world:

*I did my reserve duty without him [my service dog]. He stayed home and somehow I found myself always able to stand up straight […] as if there was a huge gap between my experience on the base, in the military framework, and outside. This feeling that here I can do anything and outside I am again in an incomprehensible world […] and from here the glitches just keep getting worse.*

The distinction between the domestic, civilian sphere and the military battleground was most characterized by the experience of order versus disorder. As outlined by participants, these tensions did not disappear upon reenlistment, rather they awaited them at the end of their service. While some tensions were familiar to the literature on reservists in general, the unique gulf between lack of functioning or disability in the domestic sphere and functioning in the military sphere was evident.

### Then and now – a remedial experience

While not directly asked to recall the traumatic experience which caused their military related PTSD, participants recalled their initial trauma with complex descriptions of failure and betrayal. Failure and betrayal was described both in reference to the specific traumatic events, as Michael described: *“Much of my trauma is from the inadequacies, ineptitude and negligence”* and also in the failure of the military to recognize their mental distress, *“and no one asked me how I was, or took interest, or clarified. And I was also a young soldier, that said to himself, this is seemingly the collective experience*” (Gary). The most central theme for participants was the discussion of the potentiality and actuality of their reserve duty during a time of war for providing a remedial experience. The remedial experience was described in a number of realms; providing a sense of capability, connection, choice and superiority, elements that were lacking in their previous, PTSD inducing, military experience.

### A remedial experience of capability

The remedial process during reserve duty was described by participants in comparison to the PTSD-inducing event that they had previously experienced in battle. Michael described how he contended with the helplessness and in turn experienced a relief in posttraumatic symptoms:

*During [reserve] service, I had no flashbacks or PTSD symptoms. On the contrary, I felt that this was the best therapy that I could ask for, because now I was able to carry all my traumas from the last time. I understood that feelings of helplessness arise from being in a situation in which we cannot win, and this time I successfully contended with these. I was able to exchange all the old helmets in my regiment for new ones, to get better equipment, bring them food and to make sure that when we went out on missions, we did this in the right way, and we trained people to do better. […] So, for me, in a strange way, the war was an amazing experience for my mental health.*

Participants described the sense of capability they experienced when they were able to control their PTSD symptoms and implement tools that learned in therapy. Rob, a 38 year old combat soldier, explained how he was able to use relaxation and grounding techniques in his daily service routine. His functioning in battle, however, was his true measure for evaluating his level of disability or ability:

*I felt it coming up and I was getting into some kind of attack. Anxiety. And I understood that this is because I recognize, I recognize the symptoms. Yes. I already know how to identify when it is imminent. And then I went, sat down someplace and did all kinds of things that I have practiced on myself to cope with this, breathing, eating or something [..] tricks that I acquired over the years, right? And while I’m doing this, something happened and an explosive device detonated on the staff of one of the companies which had been on an attack mission, and in that second, I ran to the arena to treat them. And when something like this happens you have no anxiety, I know what to do, this is what I was waiting for. I performed excellently.*

Indeed, managing symptoms and functioning at a time of acute stress provided participants with a sense of capability:

*I managed this event. So there's a kind of tension here between the fact that it's very difficult for me and it really hurts me, versus the fact that I know that in the moment of truth I'm functioning and I can save people. And that's something that was important to me. (Gary)*

Capability was also expressed as the ability to help others, in part due to having lived experience with PTSD which enabled participants to both identify those suffering and refer them for help. Andy described how: “*it’s much easier for me to identify battle reactions […] in others […]because of what I went through […] I had a type of meaningfulness, I can help others better, because of my capability, because of what I went through.”*

Assisting fellow soldiers that were experiencing acute stress symptoms was perceived by participants as a fulfillment of the knowledge they acquired about PTSD: “a*nd I saw that the younger guys were more nervous and frightened, and I saw that some of those who were my age were experiencing flashbacks and struggling a lot, and suddenly I simply felt this responsibility to be there for* (them)*”* (Michael). Thomas, a 33 year old combat soldier in charge of the evacuation of bodies described telling his comrades, *“Friends, listen up, there are unconscious bodies, there is meaning to this, because I have post trauma, lets talk about this for half a second… lets go inside (ourselves) because now we are going to see shelling and bodies”.*

The lived experience combined with a new seniority gave the participants a new role, different to that held when they experienced their initial PTSD- inducing event. Michael specifically referred the his seniority, rather than his own PTSD experience, as prompting his actions:

*I suddenly felt like, OK, you're one of the senior guys on the team. This isn't your first combat. It's on you to show the younger guys how this goes. And I saw the younger guys were nervous and scared, and I saw some of my peers were having flashbacks and struggling a lot, and I suddenly just felt this weight of responsibility to step up for everybody else. And, and that carried me through the first 2 weeks.*

This seniority was also reflected in a greater sense of meaning within their military role which contributed to a sense of purpose and capability. Peter described:

*In this sense, I felt that I was the more mature, responsible one […] So you are sort of an information base. Now you are also an information base for yourself: I know how to function in an incident like this [..] in the way of: OK, so I am now contributing to the collective, I am effective now, I am important now, I am significant now – all the things that you need to feel good.*

Finaly, the ability to support others, while highlighting new capabilities also gave participants a new role which enabled them to focus outside if their own difficulties:

*The first psychologically wounded patient arrived, and this somehow helped me, as I had to function in my role and detached me. Meaning, this is me, and that is he. He is being treated, and I am treating. That gave me a role. And I think that this allowed me to actually avoid feeling all my difficult feelings and to create for myself daily and treatment routines. (Gary)*

### A remedial experience of connection

The enhanced connection described with younger members of the team was also described in relation to a remedial experience of connection with senior military figures and institutions. Whereas the initial traumatic experiences were often described as lacking the support of superiors, the experience of returning to reserve duty often included descriptions of a new ability to mutually embrace both “macho” and “sensitive” attitudes, in part due to the acceptance of a sensitive or emotional side by seniors and mental health services, which was missing in the past.

*This war was interesting since so many guys adopted their macho side and in parallel openly embraced their feelings. And we had wonderful meetings with the “MHO’s”]mental health officers[[..] and I and some of the other guys who were in combat talked openly about our experiences and it really changed the way we spoke. They were much more open about their feelings, I think that this time the IDF learned to act, and to build a very strong, very good mental health support envelope […]” (Andy).*

The mental health support was evident both from superiors, *“I was also in constant contact with my commander […] In civilian life, he has a different profession altogether. And he's a very paternal person - he really tries to embrace and protect the department. So with him too, the feeling was very embracing.”* (Gary) and also in respect to the services provided within the military system. Despite ultimately, due to PTSD, the not continuing with reserve duty, Nick. a 29 year old combat soldier, described his experience with military mental health services when unable to function in his unit:

*[…] my officer in the department […]I was very concerned about speaking to him about this and sharing it with him and he really supported and helped me and the same with the company commander and then again with the mental health officer. He helped me in an amazing way and then after a few days he had already scheduled me a psychiatrist appointment without me knowing and simply said, go to the hospital at ten […] and I felt really treated and that they are looking after me.*

### Remedial experience by choice

The return to the battlefield with PTSD was described as one that allowed intentionality and informed choice. Informed choice was described from the decision to re-enlist through to active decisions during their service in order to protect their mental health.

The choice to re-enlist was described at varying points in the process, both at the initial outbreak but also during the period which followed. Having re-enlisted and experienced “*a very, very big anxiety attack, awful headaches, nausea, vomiting all day long”* Gary was initially sent home by his commander. He went on to describe the decision process and the choice to re-enlist to a new position:

*And I went to meet my psychologist, and we sat and really analyzed the situation, and tried to understand what would be right. And after we analyzed it, I understood that apparently it is right for me to continue participating in the war effort. To find myself the niche, the place in which I can contribute, without further injury or getting into some anxiety attack.*

Thus, the ability to make changes that allowed for continuation of service, resulted in a sense of agency, rather than failure. Choice was described as an anchor throughout the service. Peter highlighted this choice:

*And I repeat: I chose to go there, OK? I had the option. […] This means, here let’s say I say I arrived resilient, I knew where I am going. When I was in regular [army] service, it was a completely surprise incident, because everything was quiet, pastoral and beautiful, Lebanon is very lovely, OK? […] And suddenly, in a couple of seconds, the world explodes. And then you know, you are like, wait a second, but I was just in my room. […]So, the experience [of the current war] is completely different, completely different.*

### Post-trauma remedial experience – from disadvantage to advantage

In addition to the remedial experience described in relation to previous military experience, for some participants, re-enlistment was also a remedial experience for their experience with PTSD in the civilian world. Experiencing symptoms, such as hyper arousal, when not under attack can leave veterans with PTSD feeling disconnected and useless, whereas on the battlefield, hyperarousal, preparedness and a heightened sense of threat can be advantageous. Rob contended with this opposition which, in his opinion, defined logic: *“Somehow when there is an incident I know how to function, for this I practiced, this is my purpose. And the difficulty is to wait for an incident or here outside of the war, to wait for the next draft callup or to cope with the routine”*. Indeed, while acknowledging that in civilian life this arousal can be problematic, under the threat of war, for some, it was respected. Peter described from within a state of arousal:

I manage the incident because I am on it. I’m sharp, I’m sharp. And I was sharp like a mother f**ker. I would go up to my shift at four in the morning and come down at nine in the evening, not because someone is pushing me at the post, but because I choose to, OK? And I am sharp, I’m a razor. Now I’m saying this seriously. On the level of […] [who] comes to shake my hand, because he heard that I am great […] but you are really sharp. And it’s terrific. Now, in civilian life it’s less useful, OK? Less useful. Its adjacent. But when something finally happens, I think that we are the most useful – excuse me for saying so.

The remedial experience was a central theme for participants who participated in the current study. It was experienced both in terms of the behavior of the military organization and also in terms of the reservists’ performance. The gulf between functioning in previous service and in the current war was highlighted and for those who experienced a remedial experience, the experience of symptoms was also reflected upon from a stance of utility as opposed to a clinical need and hinderance to duty.

### “The intersection of fantasy with reality”

In some cases, despite a fantasy of a remedial experience, participants described limited or lack of success which led them to change positions or leave their service. While these descriptions were fewer and at times overlapped with the difficulty returning to the domestic sphere, they are included as a unique theme due to the gravity of the experience. For Andy who experienced a worsening of symptoms upon reenlistment, the impact was significant and disabling beyond his military functioning.

*Explosive noises suddenly caused me enormous anxiety. I had not had an anxiety attack like that before. I mean I did not know what was happening in my body, I didn’t know why I was so tense, why I could not think, why I could not hear others, why my pulse was so high, why I was sweating so much.*

These symptoms eventually limited his ability to stay in military service:

*And I got to a point where I could not shoot. I mean, when I shoot one shot, and my hand trembles, and I can’t think, can’t function. […] I would shoot daily even after my army service [..]I’m not…Its too much for me, it’s as if the situation is too much for me, this life is too much for me.*

Specific symptoms of PTSD were also experienced as worsening following enlistment:

*It is now even more and more difficult for me to be, ah, in closed spaces, in places where there is tumult. It was difficult before too. They are more or less the same symptoms, I just feel them more, [..] before then I had nightmares, they never ended. I always have one or so nightmares a night. Now it is every night, every night I awaken after a night of war or every morning, sometimes in early morning sometimes I don’t sleep at all. (Rob)*

The high level of functioning and meaning within the military framework, ultimately highlighted the difficulties at home as outlined in the initial theme and this, in some situations, led to a deterioration of PTSD: *“I experienced a fall, like a lack of meaning. It took me a long time until I returned to function almost normally”* (Gary, hospitalized in a rehabilitation center following discharge from reserve duty). Participants frequently concluded their interviews with a question, wondering what will be the long term impact of their service, approaching the study with an interest as to what the future holds. Indeed the unique time period of the study was reflected upon as lacking the ability to fully comprehend the impact of their service with PTSD. Peter described the unending nature of the current war: *“if you don’t have an end it’s a problem, it’s a problem”*. The temporal closeness to their reserve duty was reflected on when describing the complexity of the experience and when considering the long term effect of serving with PTSD. Having returned recently from reserve duty, Colin reflected on the utility of returning to battle with PTSD, “(as a medic, when faced with someone with a stress response) *at the end of the day it is* [the goal] *to return him to the battle. Correct or not correct? that is an excellent question”.* He concluded that on the battleground, returning to duty is to return to a familiar role and environment but that “*the difficulty with post trauma is not so much in the battle, it is in the return to routine”.* Colin ended the interview with the reflection, *“Suddenly I am capable! but I don’t know if it will last for the period after reserves. Because slowly, slowly you are returning to the same shell[…]”.* Despite not having received therapy for years, at the end of the study, Colin turned to the study coordinators for a referral for therapy.

## Discussion

The current study was born from an actuality with questions formed as the clinical reality developed. For researchers, the urgency to understand the situation was present as patients or former patients continued to enlist to reserve duty. The experience of soldiers with PTSD re-enlisting to reserve duty during a time of war was complex with difficulties contrasted with positive changes which served as remedial or restorative experiences. It was clear that the period captured in the current study was a zeitgeist and that findings were specific to the period during which the interviews were conducted. Participants had for the most part recently completed one round of reserve duty with little consideration (both on behalf of researchers and participants) dedicated to the possibility of repeated enlistment. At the time of publication, a year after the conclusion of the interviews, some reservists in Israel had recorded their fifth or sixth round of enlistment within a year and a half (Levinson, 2025). Interviews ended with questions regarding the long term impact of re-exposure and these questions remain following the conclusion of the current study. Nonetheless, participants provided novel insight into the experience of reserve duty with PTSD and were able to reflect on their experiences close to the cessation of duty.

For participants in the current study, in addition to the notable presence of those whose symptoms were exacerbated, the experience of reserve duty centered around two main themes, describing the gulf between the battleground and the home and describing the gulf between their original, PTSD inducing service and their remedial experience during the recent reserve duty.

The gulf between military duty and the transition home has been documented both in Israel (Lander et al., 2021) and the USA (Ahern et al., 2015; Cardow et al., 2021) and the experiences of the gulf between functionality on the battle ground and helplessness or disability at home for veterans returning to the military with PTSD were clearly delineated in the current study. The experience of the military as offering structure and a sense of family compared to difficulty reconnecting with family and purpose has been documented as a key stepping stone to overcome in the transition from the military to civilian life (Ahern et al., 2015). Indeed, for veterans with PTSD, social and relational functioning domains demonstrated significant correlations with symptom severity compared to occupational functioning which did not (Arenson et al., 2019). In the current study, functionality and competence on the battlefield (i.e. the occupational sphere) was compared to the lack of functioning in relational domains, specifically, in domestic life. The sense of order and roles experienced upon return to the military was welcomed in comparison to the uncertainty and chaos on the home front. Despite the military holding difficult memories, which were specifically apparent when recalling their original traumatic experiences, at a time of war, it appears that the familiarity, action and order experienced within the military framework was preferable to the helplessness, chaos and lack of action on the home front, especially when suffering from PTSD. Indeed, the response of “*I never stopped being a soldier”* highlighted the specific transitional experience for soldiers with PTSD, for many the experience of being a soldier continues through to the domestic sphere thus at times of war in particular, the return to the military was experienced as a natural return to a position which had never fully been resigned. In contrast, was the return home as a “soldier” which may present unique difficulties.

It was apparent during the Iron Swords was that there was increased attention given to the transition between home and battlefield with both mental health and behavioral science units offering transition programs with durations between hours and multiple day retreats (IDF, 2024). Based on the current findings, extended or specific programs may be appropriate to ease the transition for veterans with PTSD. While some difficulties are shared between those with and without previous diagnoses of PTSD, the current population demonstrated unique difficulties, specifically surrounding disability at home and ability on the battlefield, which may benefit from specific individual or familial processing, greater than the current level of psychological support allocated to reservists.

While the gulf between the military and domestic sphere was familiar from non-PTSD populations, the gulf most notable in the current study was that between participants’ original military PTSD inducing experience and that experienced in the current reenlistment. Without being asked a priori, participants repeatedly described their reserve duty as a reparative experience or one which significantly differed from that which triggered their PTSD response. The phrase חוויה מתקנת (literally: fixing experience) was repeatedly used and encompassed several -different realms, a sense of renewed capability, of a new relationship with superiors and the military, of choice in terms of the decision to re-enlist and where to serve and finally the conceptualization of PTSD symptoms as an advantage on the battlefield. Indications of the potential for a reparative experience initially prompted the initial study and was the dominant theme in analysis.

The descriptions of a reparative experience often were related to individualized strategies implemented to ensure the suitability of re-enlistment for the person suffering from PTSD. These included identifying symptom change, implementing a strategy (both with and without the aid of senior ranking officers) and in some cases, finding a more suitable role. This theme was reminiscent of a recent qualitative analysis of veterans experiences of Somatic Experiencing and Prolonged Exposure (Harwood-Gross et al., 2025). In this study, veterans reflected on a newfound ability to identify and regulate symptoms rather than a total extinction of PTSD response. In the current study, other than one participant who was not able to continue his military service, all participants had some experience of psychotherapy for PTSD. The language of triggers, flashbacks, regulation and preparedness indicated a familiarity with their diagnosis and participants described their ability to use their skills to assist other, younger, soldiers. Skills also appeared to be individual and varied from situation based strategies such as changing location, changing roles, recruitment of a driver through to individual strategies; breathing, grounding or consulting with their therapist. This conforms with current research findings dismissing the utility of stress management techniques (Maglione et al., 2022) and indicates the importance of individual management plans which can be implemented on and off the battlefield.

Participants reflected on their individual symptom management techniques both in reference to their own functioning but also in regard to the sense of capability garnered when they were able to notice others’ difficulties and help. The ability to help others was described as central to participants newfound capability in the military realm. Peer support in the military has been demonstrated to be beneficial for both those giving and receiving the support (Na et al., 2022) and is central to peer debriefing (Greenberg et al., 2008) and processing (Harwood-Gross et al., 2022) which replaced the debunked psychological debriefing previously used in the military (Wessely & Deahl, 2003). While many participants described ad hoc peer support, one participant (Gary) described how following a reallocation of duties, he was given responsibility for formalizing mental health support and writing a guide to help affected soldiers and their families. This formulization of the role of a PTSD sufferer within active duty was considered important. The ability for seniors and the military institution to adapt and create roles which were fitting for participants’ levels of PTSD related disability was included as part of the reparative experience and highlighted the value of an emotionally aware leadership and one which reduces mental health stigma (McGuffin et al., 2021). While the flexibility and accommodation of leadership described was for some a complete change from their initial experience of the military, it may also have been related to the surprise of the incursion and the fact that many military units were completely restructured with the unprecedented enlistment from all walks of life. Furthermore, the military described the largest recruitment of mental health officers during the current war (Klein, 2024) which may have impacted the level of mental health awareness experienced by current participants.

Accommodation by the military to new or adapted roles or to enable greater emotional support, the sense of capability and adaptiveness of PTSD symptoms and clear roles and sense of purpose contributed to the reparative experience described. Furthermore, even when functioning was impaired and discharge of duties imperative, the potential for reparative experience was still present with the manner of referral and hand holding experienced as a positive experience (mental health officer booking a psychiatrist appointment and providing immediate mental health services). Additional to the role of the military in the reparative experience, participants’ experiences highlighted several factors which appeared to be instrumental in the experience. The ability to reconceptualize symptoms as adaptive or advantageous and respond to those which impacted functioning demonstrated a high level of psychological awareness one which should not be assumed for soldiers re-enlisting with PTSD. Furthermore, the sense of capability was enhanced by re-enlistment to a more senior or respected position even if this change was only a factor of age and not rank. Seniority was experienced both as a function of increased coping abilities and a sense of maturity and confidence but was also reinforced by those in the environment. As these key elements must be noted and reflected upon for future enlistments, so too must re-enlistment be questioned when these accommodations or roles are not possible.

In addition to the theme of reparation and the positive impact of re-enlistment, so too did the reservists question the negative impact of their service. Participants frequently questioned the effect of reenlistment on their civilian life and reflected a fear, now also propagated by the media (Levinson, 2025). For one participant in the current study, they experienced the recurrence of PTSD symptoms, which seemingly had been simmering at the time of enlistment, and their increase caused a total lack of functioning prior to entering the warzone. For others the experience of a worsening of symptoms upon return to civilian life led to difficulties in functioning, which in some cases led to a need to resume psychological treatment or a breakdown in marital functioning. For most participants, even when re-enlistment was experienced as a total extinction of symptoms, they asked themselves how long this could last or whether they would pay a price in other realms. Participants questioned the generalizability of their experience and researchers questioned findings as the war continued and the time spent in reserve duty was extended.

As outlined thus far, participants highlighted both the effort invested by themselves and the military in order to ensure a successful service and the price they were willing to risk in order to re-enlist. The participants’ in the current study spoke not only of their service as being of importance to the war effort rather of importance to them as individuals. These findings must be considered both in respect to the specific time-period, the outbreak of sudden war, but also the militarized culture present in Israeli society and the conjunction of militarized masculinity as shaping individual identities. For many reasons, including compulsory national conscription and ongoing military battles, military culture has shaped Israelis’ perceptions of masculinity, good citizenship and social class (Levy & Sasson-Levy, 2008). As Levy and Sasson-Levy outline, the militarization of society in Israel begins long before enlistment, with military service considered the hallmark of good citizenship and the antithesis to the decimation of the Jewish people experienced in the Holocaust prior to the establishment of the Jewish State. It is this socialization from a young age which penetrates Israeli society and can be exemplified in current participants’ ongoing relationship to the military or lack of complete detachment following their diagnosis of PTSD. Indeed for many of the participants in the current study, the opportunity for a reparative experience and return to the military was in coherence with the Israeli narrative of a hero. The memorialization and exaltation of soldiers who die in the line of duty and glorification of acts of valor and strength is in coherence with the image of the “New Jew”, created with the State of Israel and tasked with ensuring national survival (Mayer, 2012) and the antithesis of the Jew of the ghetto or, in relation to the current population, the lack of functioning which is characteristic of experience of PTSD.

*When national survival is attributed almost exclusively to the heroism of warriors, nationalism and masculinity become inseparable. And when a nation cultivates its own myths of survival, recalls the struggles of its past, and celebrates its heroes, it perpetuates the intimate tie between nation and male and continually constructs the image of both its desired nationalism and its desired son. This son, in whose image the younger generation is socialized, is most dear to the nation when he takes up arms and willingly risks his life for the survival of the nation and when he wins military battles on the nation’s behalf*. (Mayer, 2012, p. 339)

When Israel was invaded on the 7th of October 2023, the dilemma of taking up arms and risking life was even more pertinent for those who’s previous wars and battles still haunted them. As the current study demonstrated, for many, the decision to re-enlist was impacted by the acute sense of danger and threat faced by them and their families but so too was the national narrative of militarized masculinity, valour and worth, a societal backdrop. When the current participants decided to take up arms and re-enlist, they had their personal narrative which was reflected in the interviews but it is impossible to detach from the national narrative which permeates society.

The current study captured a specific time period but also captured the potential experience of the two percent (or more) worldwide who re-enlist to military service with PTSD. The study was limited by its small sample size and unique setting, though in part investigated a similar phenomenon to that witnessed amongst soldiers taking part in the Ukraine-Russia war who both experience the direct attack on their homes and have been reported as returning to combat with PTSD (Khalilova, 2024). Many participants in this study demonstrated a degree of reflective ability. The ability for participants to reflect on their own psychological skills for coping with flashbacks or anxiety and the accommodations which were made to enhance their service indicated a level of psychological health. While participants reflected on difficulties during service, in some cases returning home and then returning to the military, most also demonstrated a level of functioning which enabled them to be active parts of their unit. It is important to consider that many reservists with and without PTSD were referred for mental health support and for the development of PTSD following exposure to combat during the period of the study and it is unknown how many were previously diagnosed with PTSD (Shelef et al., 2025). This study must not be used as evidence that the military should actively recruit reservists with PTSD rather how better to support those who have chosen to re-enlist. Further research is crucial to understand the scale of re-enlistment with PTSD and the impact of re-exposure to combat, specifically amongst those who were not able to function within their unit or who’s psychological wellbeing significantly deteriorated following discharge. Furthermore, as the study was written up, and the magnitude of the war unfolded, the current research team noted how the interpretation of findings was approached with greater caution about extrapolation. Researchers noted increased psychological difficulty presenting in the clinic and in the public sphere and increased concern as to the viability of the current findings for extended periods of reserve duty. In order to understand the experience of reservists with PTSD who re-enlisted into reserves, it is crucial to conduct interviews at varying time points and amongst a wider sample, specifically a sample comprised of those who had less successful experiences.

## Conclusion

The decision to re-enlist with PTSD is complex, both from the viewpoint of the veteran and the motivation to return to the scene of the trauma, and from the decision of the military to re-enlist someone with an emotional vulnerability. At a time of unexpected war these decisions become even more complicated and at times removed from consideration due to the sudden and extreme need for manpower. The current study examined the unique stories of reservists as they narrated their experience returning to the military with PTSD. The experience was characterized by the difference between the helpless and chaotic scene at home versus the familiarity and order which returning to the military could provide. Participants primarily told of a reparative experience encompassing multiple elements but also raised questions about the long term potential or detriment of re-enlistment. The positive experiences described by reservists with PTSD may be in part due to the ability to manage symptoms and process future trauma exposure using skills garnered from previous psychological work. Further research is needed to assess the long-term impact of re-enlistment with PTSD and amongst a wider population including those without such an ability to process their experience.

## Declaration of competing interest

The authors declare that they have no known competing financial interests or personal relationships that could have appeared to influence the work reported in this paper.

## Appendix A

Interview Guide (Harwood-Gross et al.)

General Background

"Tell me about what it was like for you to be a reserve combat soldier with post-trauma."

(Additional prompt to understand PTSD relevance: Tell me the story of your coping with post-trauma, from the moment you were diagnosed until your most recent reserve service.)

**Questions About Reserve Service**

1. Enlistment Experience

• Tell me about your experience when being called up for reserve duty.
• Tell me about the decision to enlist for reserve duty.
• Were there others involved in the decision to enlist? If so, please tell me about that.
• Try to recall the moment of enlistment itself. Can you tell me about it? [For interviewer: Ensure reference to feelings, thoughts, and key experiences].

2. Meaningful Moments

• Try to think about moments and/or meaningful events you had during reserve duty. Can you tell me about them? [For interviewer: Ensure that the interviewee shares several events of different types].

3. Impact of PTSD on Reserve Service

• How did the PTSD affect your emotions? Tell me a story about a moment when this impact was significant.
• How did the post-trauma affect your behaviors? Tell me a story about a moment when this impact was significant.
• How did the post-trauma affect your thoughts during service? Tell me a story about a moment when this impact was significant.

4. Impact of Reserve Service on PTSD

• Can you share with me the impact that reserve service had on your experience of post-trauma? Tell me a story about a moment when this impact was significant. [For interviewer: If the topic does not arise, ask about the effect of service on specific symptoms mentioned in the background question].

5. Overall Experience

• Tell me how, in your view, the post-trauma influenced your reserve service. Tell me a story about a moment when this influence was significant.
• Looking back, how would you describe your reserve service experience? [For interviewer: Check if the interviewee sees a connection between the overall reserve experience and being diagnosed with post-trauma].

6. Reflections and Insights

• In light of reserve service, do you have new understandings regarding the post-trauma you suffer from? Tell me about it.
• In light of this current service, do you have new understandings regarding your previous military service? Tell me about it.

Is there something important that should be known for others coping with similar issues?
